# Supplementary material for: Resolving the full spectrum of human genome variation using Linked-Reads
Source: Genome Res. 2019 Apr;29(4):635–45. doi: 10.1101/gr.234443.118 (PMC6442396; doi:10.1101/gr.234443.118)
Supplement: Supplemental Material [file supp_gr.234443.118_Supplemental_longranger-2.2.zip › longranger-2.2.tar.bz2!/lr2.2-public/lib/stan/cmdstan/stan/doc/api/html/modulus_8hpp_source.html]

Stan: src/stan/math/functions/modulus.hpp Source File


|  |  |
| --- | --- |
|  | Stan  2.5.0  probability, sampling & optimization |


- Main Page
- Related Pages
- Namespaces
- Classes
- Files

- File List
- File Members

All Classes Namespaces Files Functions Variables Typedefs Enumerations Enumerator Friends Macros Pages

- src
- stan
- math
- functions

modulus.hpp

Go to the documentation of this file.

1 #ifndef STAN\_\_MATH\_\_FUNCTIONS\_\_MODULUS\_HPP

2 #define STAN\_\_MATH\_\_FUNCTIONS\_\_MODULUS\_HPP

3

4 #include <vector>

5 #include <cstddef>

6 #include <cstdlib>

7

8 namespace stan {

9  namespace math {

10

11  inline int modulus(const int x, const int y) {

12  return std::div(x, y).rem;

13  }

14

15  }

16 }

17

18 #endif

stan::math::modulus

int modulus(const int x, const int y)

**Definition:** modulus.hpp:11


---

[ Stan Home Page ]

*© 2011–2014,
Stan Development Team.*
